# Management of healthcare areas for the prevention of COVID-19 emergency in an Italian teaching hospital in Pisa, Tuscany: A hospital renovation plan

**DOI:** 10.1017/ice.2020.177

**Published:** 2020-04-27

**Authors:** Angelo Baggiani, Silvia Briani, Grazia Luchini, Mauro Giraldi, Maria Carola Martino, Andrea Porretta, Michele Totaro, Gaetano Privitera

**Affiliations:** 1 Hygiene and Epidemiology Unit, Azienda Ospedaliero Universitaria Pisana, Pisa, Italy; 2 Department of Translational Research and the New Technologies in Medicine and Surgery, University of Pisa, Pisa, Italy; 3 General Management Unit, Azienda Ospedaliero Universitaria Pisana, Pisa, Italy; 4 Health Management Unit, Azienda Ospedaliero Universitaria Pisana, Pisa, Italy; 5 Hospital Medical Direction Unit, Azienda Ospedaliero Universitaria Pisana, Pisa, Italy; 6 Disaster Manager Unit, Azienda Ospedaliero Universitaria Pisana, Pisa, Italy

*To the Editor—*The current outbreak of coronavirus disease 2019 (COVID-19), which began in Wuhan, China, in December 2019, has spread to >160 countries in <3 months. In March 2020, the World Health Organization (WHO) defined this outbreak as a pandemic emergency that requires strategies to manage the infectious risk, including social distancing measures and border closures.^1,2^

In Italy, a cluster of cases began in Lombardy on February 21, and by March 1, the virus had spread to all Italian regions. From February 21 to March 24, a total of 69,176 cases were confirmed, with 6,820 deaths. The Tuscany region has registered 2,699 cases with 918 hospitalized patients. Of these 918 patients, 244 (27%) had respiratory failure and required specialized care in an intensive care unit (ICU).^3^ Considering the limited number of beds in ICUs, we describe the technical emergency measures adopted by a 1,081-bed teaching hospital located in the Pisa district (Tuscany, Italy) that aimed to prevent hospital overcrowding by COVID-19 patients.

Following the first confirmed COVID-19 case in Tuscany on February 25, a task force assembled with representatives from several hospital areas: medical direction, hygiene and epidemiology, facility management, occupational medicine, emergency medicine, and intensive care units. After drafting a procedure for COVID-19 patient management, the working group divided the hospital and emergency department into COVID and not-COVID areas organized into specific emergency departments, medical wards, ICUs, and operating rooms (Table 1).

**Table 1.** Rational Division of Hospital in COVID and Non-COVID Areas

Emergency Medicine Hospital			
COVID Area			Non-COVID Area
Emergency department Pre-triage area Triage area Negative pressure room Swabbing Room			Emergency department Triage area
Medical wards^a^ Infectious disease unit (23 beds) Pulmonology Unit (19 beds) **COVID area 1 (36 beds)** **COVID area 2 (36 beds)** **COVID area 3 (54 beds)** **COVID area 4 (34 beds)**			Medical wards 108 beds
ICUs	Normal Beds	C-PAP Beds	ICUs 38 beds
ICU-1	14	0	
**ICU-2**	**7**	**6**	
**ICU-3**	**10**	**12**	
**ICU-4**	**10**	**12**	
**ICU-5**	**12**	**0**	
Operating rooms 3 rooms			Operating rooms 23 rooms
Old hospital **30 beds**			

Note. ICU, intensive care unit; C-PAP, continuous positive airway pressure.

a New beds in medical wards and ICUs are shown in bold.

As part of the new procedure, healthcare workers do not move from one area to another. In non-COVID areas, patients and workers use surgical mask, gloves and gowns as personal protective equipment (PPE) as described by the World Health Organization.^4^

In COVID areas, FFP2 or FFP3 masks, eye protection, a double pair of gloves and a second gown are recommended during the aerosol-generating procedures (eg, swabbing, C-PAP therapy, etc). All masks must be certified according to the European standard (BS EN 149:2001).^5^

Considering the lack of sufficient beds in medicine wards and ICUs, the working group started a program to enlarge these areas. Beginning on March 19, several medical wards and operating rooms, located in different hospital units, have been repurposed to create new 160 COVID beds in medical wards (ie, COVID areas 1–4) and 69 COVID beds in ICUs (ie, COVID ICUs 2–5). In a old and previously disused hospital, a new area with 30 beds has been activated. Negative-pressure isolation room systems (−9 Pa) and high-efficiency particulate air (HEPA)–15 filters were installed in COVID ICUs to contain airborne microorganisms within the rooms. Negative-pressure isolation rooms are commonly used for COVID-19–positive patients with respiratory failure because they are usually treated with aerosol-generating C-PAP therapy (ie, they occupy C-PAP beds).^6^

On February 25, the hygiene and epidemiology unit and the prevention-protection service began providing telematic training to all hospital workers. Related course work has been developed to address various issues. A preliminary lesson was prepared on the epidemiological occurrences related to β-coronavirus outbreaks, with specific references to SARS-CoV-2. Furthermore, specific instruction on the importance of hospital disinfection, hand hygiene, and the use of PPE, has been provided to all healthcare personnel (ie, physicians, nurses, cleaner workers, etc). Finally, additional technical training has been provided to maintenance workers responsible for aeraulic systems to enhance their performance quality during this pandemic.

In conclusion, the emergence of the COVID-19 pandemic has required synergic cohesion of the working group to define the principal risks for patients and healthcare workers and to implement preventive measures such as PPE and training courses. At the same time, rapid renovation work in a hospital nearing completion was undertaken to enlarge the ICU areas for patients with respiratory failure. Overall, the structural division into non-COVID and COVID areas could be the best precautionary strategy to avoid the infectious risk between patients and staff.
